# Dielectric Properties of Composite PZT Films with Distinct Phase-Transition Temperatures via Aerosol Deposition

**DOI:** 10.3390/ma18071427

**Published:** 2025-03-24

**Authors:** Ye-Ji Son, Seung-Wook Kim, Hyo-Min Kim, Hyojung Kim, Baojin Chu, Dae-Yong Jeong

**Affiliations:** 1 Program in Semiconductor Convergence, Department of Materials Science and Engineering, Inha University, 100 Inha-ro, Michuhol-gu, Incheon 22212, Republic of Korea; 20191063Son@inha.edu (Y.-J.S.); sw_kim_@naver.com (S.-W.K.); norihm@inha.edu (H.-M.K.); 2 Department of Semiconductor Systems Engineering, Sejong University, 209, Neungdong-ro, Gwangjin-gu 05006, Republic of Korea; hyojungkim0912@sejong.ac.kr; 3 Department of Materials Science and Engineering, University of Science and Technology of China, Hefei 230026, China

**Keywords:** lead zirconate titanate, aerosol deposition, phase coexistence, morphotropic phase boundary, temperature stability

## Abstract

With the increasing demand for ceramic-based capacitors in energy storage and electronics, ferroelectrics have gained attention due to their high dielectric coefficient. However, near the phase-transition temperature, a significant variation in dielectric coefficient leads to reduced temperature stability and degradation of electrical properties, limiting their applications. To address this, composite films with multiple phase-transition temperatures can provide a stable dielectric response over a broad temperature range. Conventional ceramic processing cannot achieve this due to interdiffusion during high-temperature sintering. To overcome this, we utilized the aerosol deposition (AD) process, which enables the fabrication of high-density ceramic films at room temperature while preserving the distinct Curie temperatures (*T_c_*) of different compositions. We prepared composite films with three PZT compositions: Pb(Zr_0.2_Ti_0.8_)O_3_, Pb(Zr_0.52_Ti_0.48_)O_3_, and Pb(Zr_0.8_Ti_0.2_)O_3_. Compared to single-phase Pb(Zr_0.52_Ti_0.48_)O_3_, the composite film exhibited a higher dielectric coefficient with reduced variation across a broad temperature range due to overlapping phase transitions. The AD-fabricated composite PZT films offer enhanced thermal stability, making them suitable for temperature-sensitive applications such as compact power electronics and portable devices.

## 1. Introduction

Lead zirconate titanate (PZT: PbZr_x_Ti_(1−x)_O_3_) is a material of great interest due to its remarkable property enhancements in the morphotropic phase boundary (MPB) region, making it highly suitable for various applications. In the MPB region, tetragonal and rhombohedral phases coexist, maximizing interactions between the phases. Notably, polycrystalline PZT ceramics near the MPB composition of x ≈ 0.52 exhibit maximum dielectric permittivity and electromechanical coupling coefficients, which enhance their potential for use in diverse applications, such as piezoelectric transducers, pyroelectric detectors, and non-volatile memory devices [1]. It maintains a relatively stable dielectric coefficient up to approximately 200 °C; however, beyond this temperature, a phase transition occurs within a narrow temperature range, causing a sharp increase in the dielectric coefficient and pronounced temperature dependence. Analyzing the temperature-dependent permittivity data of conventional PZT bulk reveals the following trends. In the temperature range of 25 °C to 200 °C, prior to entering the phase-transition region, the permittivity increases relatively moderately, with a change rate of approximately 15%. However, in the range of 200 °C to 300 °C, approaching the phase-transition region, the permittivity increases sharply, resulting in a significant change rate of around 86%, which coincides with the rhombohedral-to-tetragonal phase transition, further intensifying permittivity variation. Particularly, in the range of 300 °C to 350 °C, as the material enters the phase-transition region, the permittivity change rate dramatically rises to approximately 107% [2]. This rapid change in permittivity reflects the structural transformations and sensitivity of domain alignment within the phase-transition region. Despite the high absolute values of permittivity, the excessively large relative change rate with temperature imposes limitations on its reliability for the broad temperature range. Therefore, strategies to suppress or stabilize the permittivity change rate in the phase-transition region are essential for improving the practicality of these materials with thermal stability. Therefore, the key is to maintain high permittivity over a wide temperature range, even at high temperatures where phase transitions occur, while minimizing the variation in permittivity with temperature.

Doping has traditionally been used to increase dielectric stability at high temperatures, but it often lowers the Curie temperature (*T_c_*), narrowing the temperature range where high permittivity can be maintained. Additionally, doping can introduce structural instabilities or amplify thermal property variations, which may degrade dielectric performance in high-temperature environments [3,4,5]. To address these limitations, the present study proposes a method that utilizes compositions near *T_c_* to maintain high permittivity while mixing compositions with different *T_c_* values to broaden the phase-transition temperature range. By combining the different compositions, a wide temperature range with phase coexistence was achieved, effectively smoothing variations in dielectric properties and maintaining stable, high permittivity without relying on dopants.

In general, ceramic fabrication requires high temperature to activate the chemical reaction and diffusion for densification. AD is an innovative process that enables the formation of high-density ceramic films at room temperature, preventing phase changes or material degradation caused by heat [6]. For an AD, the powder is aerosolized and mixed with gas, then accelerated at high speed through a nozzle to impact the substrate, forming a thick film with the exact same composition of powders. Notably, AD prevents heat-induced phase transitions, making it particularly suitable for applications where high-temperature processes are not feasible. Traditional high-temperature sintering often leads to the synthesis of new phases, disrupting the mixed state of materials. In contrast, the AD method allows for room-temperature densification, effectively preserving the mixed state and preventing the chemical reaction, which is critical for ensuring the composite films [6].

For composite film, three compositions with different phase-transition temperatures, namely Pb(Zr_0.52_,Ti_0.48_)O_3_ (PZ5T5), Pb(Zr_0.2_,Ti_0.8_)O_3_ (PZ2T8), and Pb(Zr_0.8_,Ti_0.2_)O_3_ (PZ8T2), were selected. The three compositions were mixed in equal proportions to create a homogeneous blend, which was then used to fabricate composite films using the AD technique. We hypothesized that the coexistence of various phases occurs in a metastable state rather than a thermodynamic equilibrium. The composite films with this metastable state allow gradual phase transitions, enabling a high dielectric coefficient for a broad temperature range. The fabricated films were subsequently subjected to electrical property measurements, focusing on analyzing the changes in permittivity across a wide temperature range to evaluate their performance.

## 2. Experimental Procedure

For the AD process, three kinds of powder with different compositions of Pb(Zr_0.8_,Ti_0.2_)O_3_ (PZ8T2), Pb(Zr_0.52_,Ti_0.48_)O_3_ (PZ5T5), and Pb(Zr_0.2_,Ti_0.8_)O_3_ (PZ2T8) were synthesized through a solid-state reaction method using PbO, ZrO_2_, and TiO_2_ (99.9% purity, Sigma-Aldrich, Saint Louis, MO, USA). The powders were mixed with ethyl alcohol (99 purity, Duksan, Seoul, Republic of Korea) and subjected to wet ball milling for 24 h to ensure homogeneous mixing. The milling process was conducted at a rotation rate of 300 rpm using zirconia balls as the milling media, and a plastic bottle was used as the milling container. After mixing, the mixed powders were calcined at 800 °C for 4 h to promote phase formation. The calcined powder was then sieved to remove any agglomerates and ensure uniform particle size. The prepared PZT powders were mixed in equal ratios and wet-ball-milled for 24 h to achieve a homogeneous blend suitable for the AD processing. After the second milling, the powder was sieved again to ensure uniform particle size distribution. The prepared Pb(Zr_0.8_,Ti_0.2_)O_3_ (PZ8T2), Pb(Zr_0.52_,Ti_0.48_)O_3_ (PZ5T5), and Pb(Zr_0.2_,Ti_0.8_)O_3_ (PZ2T8) and composite PZT powders were fabricated in the form of films by the aerosol deposition process on Pt-coated Pt(111)/Ti/SiO_2_/Si(100) substrates. The AD process was conducted with changing MFC values, as detailed in Table 1. After the AD process, the films were annealed in a box furnace (Furnace 23ltr CWF11/23/PID301, Carbolite Gero, Hope Valley, UK) at 550 °C for 2 h. The heating and cooling rates were both set to 5 °C/min to enhance crystallinity and relieve internal stresses developed during the deposition process.

Despite slight variations in the AD conditions, the overall elemental composition of the films remained consistent within an acceptable range, as confirmed by XRD and EDX analysis. These variations did not have a significant impact on the compositional uniformity or the observed properties, ensuring the reliability of the results.

The crystal structure and cross-sectional morphology of the PZT films fabricated through the AD process were examined using the following analyses. First, high-resolution X-ray diffraction (HR-XRD; Philips X’pert Pro MRD Diffractometer, Philips, Amsterdam, The Netherlands) was employed to analyze the crystallinity of PZT powders and AD films, ensuring that the three compositions were well mixed and properly formed into a film. The XRD measurements were conducted using a Cu-Kα radiation source (λ = 1.5406 Å) with a scanning speed of 5°/min over a 2θ range of 20–80°. Additionally, a field emission scanning electron microscope (FE-SEM; S-4300SE, Hitachi, Tokyo, Japan) was used to observe the cross-sectional morphology, confirming the formation of a uniform, dense film with a thickness of 9 µm.

To evaluate the electrical properties of the PZT films, Pt top electrodes with a diameter of 0.5 mm were formed on the surface of the films using a DC sputtering system (Crossington108 auto sputter coater). Dielectric properties, including the dielectric coefficient (ε_r_) and dielectric loss, were measured with an impedance analyzer (Agilent Technologies 4194A, Santa Clara, CA, USA) while heating at a rate of 5 °C per minute. Additionally, ferroelectric characteristics were investigated by obtaining P-E hysteresis loops using a precision multiferroic and ferroelectric test system (P-PMF-K; Radiant Technologies, Albuquerque, NM, USA). To ensure consistent temperature-dependent dielectric measurements, the heating rate was controlled at 5 °C/min using a PID-controlled furnace (Lindberg/Blue M, Thermo Scientific, Waltham, MA, USA).

## 3. Results and Discussion

**Figure 1:**
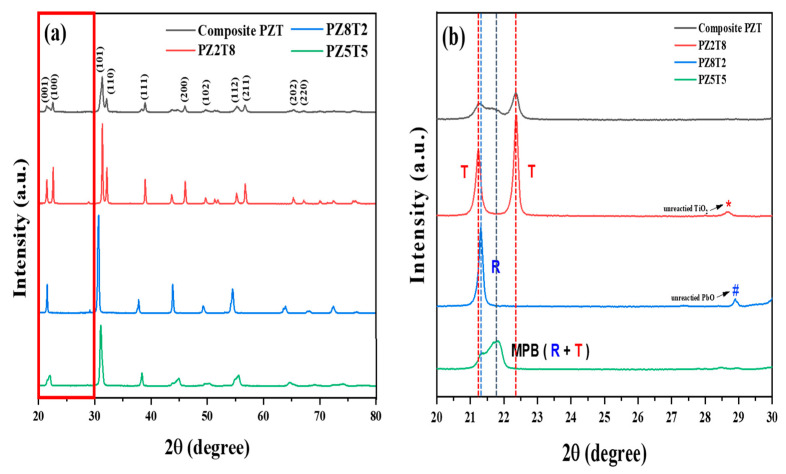
(**a**) XRD patterns of PZ8T2, PZ5T5, PZ2T8, and composite PZT powders in the 2θ range of 20–80°, with the region from 20° to 30° highlighted for detailed analysis. and (**b**) PZ8T2, PZ5T5, PZ2T8, and composite PZT powders in the 2θ range of 20–30° (* is TiO_2_; # is PbO).

XRD patterns from 2θ = 20 to 80° are shown in Figure 1a, which shows that the high-intensity peaks for each composition correspond to specific crystallographic planes labeled in the XRD patterns, such as (100), (110), (111), and (200). This indicates that all samples have a consistent perovskite structure, with slight shifts in peak positions due to the differences in Zr/Ti ratios. These differences reflect the variation in the *T_c_* of each composition. The XRD patterns enlarged in the 2θ range of 20° to 30°, as shown in Figure 1b, reveal the crystallographic phases of the PZT compositions. PZ8T2 exhibited characteristic peaks corresponding to the rhombohedral (R) phase, while PZ2T8 showed peaks associated with the tetragonal (T) phase. Here, the small peaks around 29° in PZ8T2 and PZ2T8 were indexed to the unreacted PbO and TiO_2_, respectively. However, after AD process, these unreacted components were not detected in the AD film due to the chemical reaction during AD and annealing process [7]. PZ5T5 corresponds to the MPB (morphotropic phase boundary) region and showed an MPB peak that reflects the coexistence characteristics of the R and T phases. In particular, the peaks observed in the 2θ range of 21° to 23° confirm that the peaks of PZ5T5, PZ8T2, and PZ2T8 are all included within the peaks of the PZT mixture powder. Additionally, the PZT mixture powder exhibited a smoother and more evenly distributed set of peaks, indicating that successful mixing and homogenization were achieved through the mixing process.

**Figure 2:**
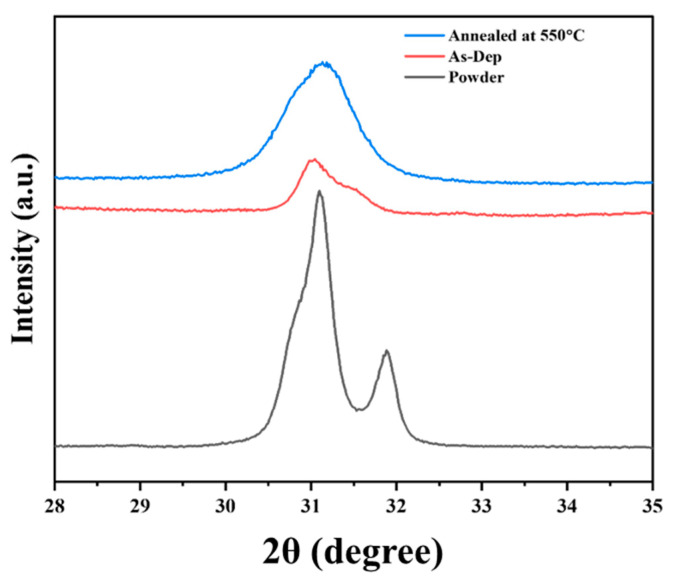
XRD patterns of composite PZT powders, as-deposited film, and AD film annealed at 550 °C.

To evaluate the effect of post-deposition annealing on crystallinity, the XRD patterns of PZT powder, as-deposited AD film, and annealed PZT thick film at 550 °C are compared in Figure 2. The peaks observed between 30° and 33° highlight the changes in crystallinity before and after annealing. The as-deposited AD film shows an amorphous tendency with reduced crystallinity, primarily due to structural distortion caused during the AD process. This distortion arose from the high-speed impact of particles during deposition, which prevents proper crystal recovery and alignment. After annealing the composite PZT film at 550 °C, a significant increase in peak intensity can be observed in the XRD pattern, indicating increased crystallinity. The thermal energy supplied during the annealing process effectively relaxed the distorted crystals, aligned the crystal structure, and stabilized the overall crystalline framework [8].

**Figure 3:**
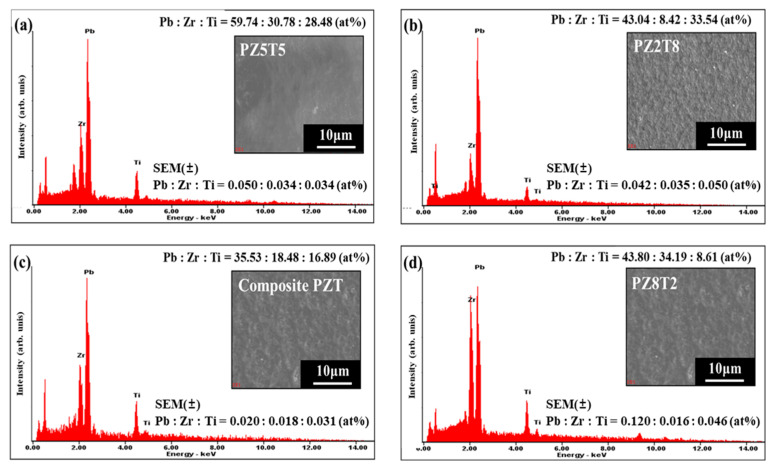
Elemental Mapping and EDX Spectrum of AD-Processed (**a**) PZ5T5, (**b**) PZ2T8, (**c**) composite PZT, and (**d**) PZ8T2 Films.

Figure 3 shows the EDX elemental composition analysis of annealed PZT films and standard error of measurement (SEM). The EDX spectra of (a) PZ5T5, (b) PZ2T8, (c) composite PZT, and (d) PZ8T2 films after annealing at 550 °C confirm the presence of Pb, Zr, Ti, and O, with elemental ratios closely matching the theoretical composition. In (a), (b), and (d), the quantified elemental composition of each film demonstrates that the PbO, ZrO_2_, and TiO_2_ ratios remained consistent after deposition and annealing. In (c), the EDX analysis of the composite PZT film, synthesized by mixing the three compositions, shows a uniform elemental distribution, aligning with the expected stoichiometry.

**Figure 4:**
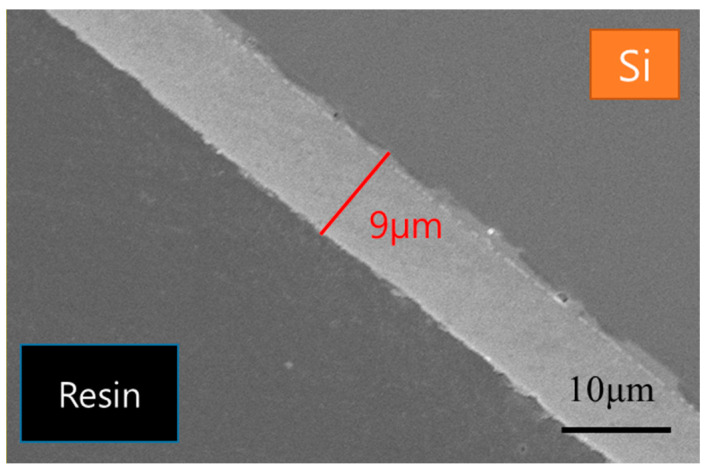
SEM cross-section image of the as-deposited PZT film with a thickness of 9 µm.

The cross-sectional SEM image in Figure 4 reveals that the AD process successfully produced a dense film with a thickness of 9 μm, showing no visible porosity and confirming the successful execution of the synthesis and deposition processes. These results demonstrate that the thick film was uniformly formed with a dense structure, meeting the material properties required for subsequent studies.

**Figure 5:**
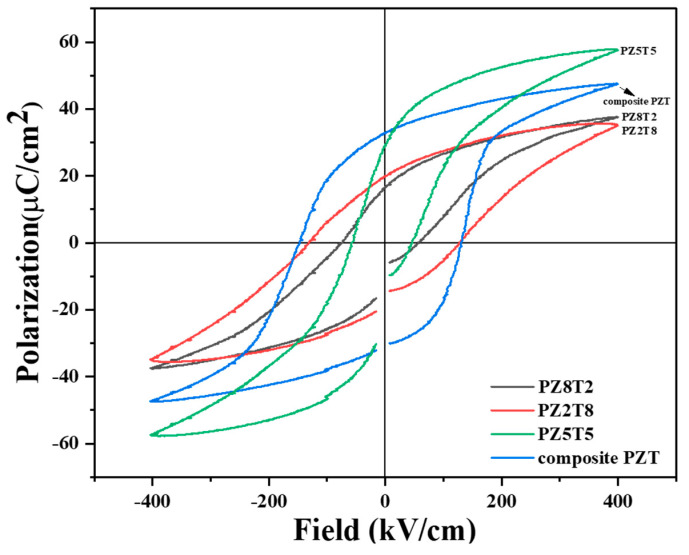
Polarization–electric field hysteresis loops of film types PZ5T5, PZ2T8, PZ8T2, and composite PZT compositions fabricated by aerosol deposition method (measured at 100 Hz).

The polarization behavior of the PZT films under an applied electric field was analyzed using hysteresis loops, which provided valuable insights into their ferroelectric properties. The polarization-electric field (P-E) hysteresis loops presented in Figure 5 reveal distinct differences in ferroelectric characteristics among these compositions. All PZT films were conducted at a frequency of 100 Hz. Table 2 also shows the numerical values of remnant polarization (P_r_), saturated polarization (P_s_), and cohesive field (E_c_) for each PZT composition film.

The P_r_ value of PZ2T8 was 16.13, while that of PZ8T2 was 19.61. In the case of PZ2T8 with a tetragonal phase, a more significant change in polarization was observed compared to the rhombohedral-phase PZ8T2, resulting in relatively higher remanent polarization (P_r_) values. Additionally, for the PZ5T5 composition, the P_r_ value was 28.20, and the P_s_ value was 57.77, indicating a significant increase in both spontaneous polarization (P_s_) and remanent polarization (P_r_) in the hysteresis loop. This enhancement can be attributed to the dielectric increasement effect within the morphotropic phase boundary (MPB) region.

Compared to these three individual composition, the composite PZT composition exhibited the highest remanent polarization (*P_r_*) value of 32.91. The high remanent polarization (*P_r_*) observed in the composite PZT film can be explained by the structural characteristics of the mixed composition and the effects of the MPB region, which is artificially driven [9,10]. In the MPB region, the coexistence of rhombohedral and tetragonal phases facilitates domain switching, and these structural properties help maintain a stable polarization state even after the external electric field is removed. The maximum polarization (P_max_) value of the composite PZT film was 47.44, which is lower than the P_max_ value of 57.77 for the single-phase PZ5T5. However, the composite PZT film still exhibited significantly high polarization and distinct ferroelectric properties. As the composite PZT film is composed of three different compositions, it can be expected that the properties of composite PZT film were the sum of each composition. However, during the AD and annealing process, the three compositions reacted to each other, and the final complex composition and microstructure gave the final P-E behavior and dielectric property change with temperature. The coercive field can increase as the ferroelectric phase is formed and stabilized by the annealing process in a composite structure with heterogeneous phases mixed together, and the domain alignment is strengthened during the stabilization of multiple phases, which can induce domain transitions that require high electric fields [11].

**Figure 6:**
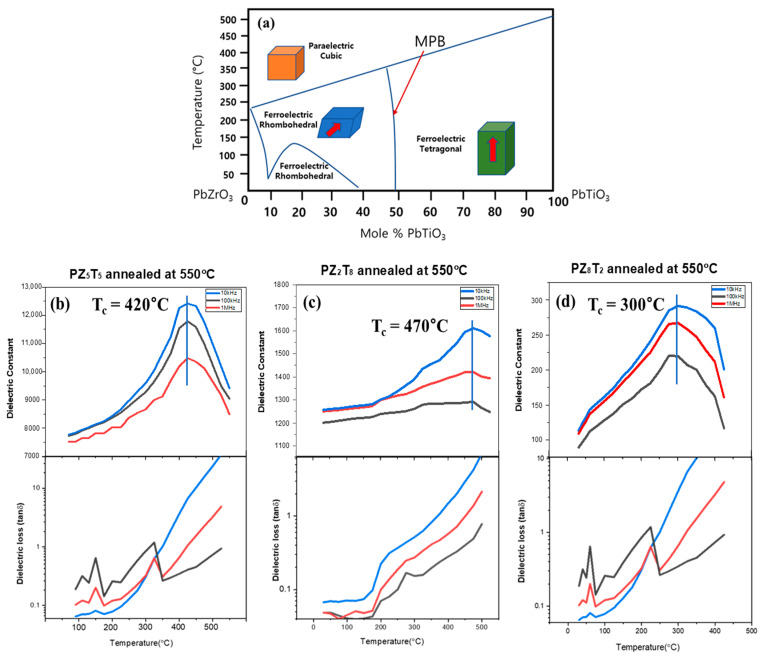
(**a**) PZT phase equilibrium diagram and dielectric coefficient and dielectric loss of (**b**) film-type PZ5T5, (**c**) film-type PZ2T8, and (**d**) film-type PZ8T2 as a function of temperature at various frequencies, all fabricated using the aerosol deposition (AD) method and annealed at 550 °C.

Figure 6 illustrates the temperature-dependent changes in the dielectric coefficient and dielectric loss of PZT compositions, including PZ5T5, PZ2T8, and PZ8T2, measured at different frequencies.

Figure 6a illustrates the phase diagram of PZT, highlighting three key compositions: PZ (PbZrO_3_-rich composition, denoted as rhombohedral), the MPB (morphotropic phase boundary) region, and PT (PbTiO_3_-rich composition, denoted as tetragonal) [11]. The *T_c_* of these compositions are marked with dashed lines and blue stars, indicating the temperatures at which phase transitions occur. The MPB region in PZT is a range where the rhombohedral (R) and tetragonal (T) phases coexist, maximizing the interaction between the two phases. In this region, polarization directions become more diverse, making the material highly responsive to electric fields, which is a key factor in achieving high dielectric and piezoelectric properties. These characteristics make the MPB region a critical element in realizing the exceptional electromechanical performance of PZT. The material transitions to the cubic phase (C) at each *T_c_*, where dielectric properties significantly change. On the left, PZ8T2 represents the rhombohedral phase (R), while the PZ5T5 MPB region exhibits the coexistence of rhombohedral and tetragonal phases, offering unique dielectric and piezoelectric properties. On the right, PZ2T8 represents the tetragonal phase (T). This diagram provides a clear representation of how the phase behavior and transitions vary with temperature and composition, emphasizing the formation of the cubic phase beyond the *T_c_*.

Figure 6b–d show the temperature-dependent dielectric coefficient and dielectric loss of film-types PZ8T2, PZ5T5, and PZ2T8 fabricated using the AD method. In Figure 6b, the dielectric coefficient of PZ5T5 increases with temperature, peaking at the *T_c_* (420 °C), then decreases as temperature rises further. Similarly in Figure 6c, the dielectric coefficient of PZ2T8 also increases with rising temperature, reaching its maximum at the *T_c_* (470 °C), and decreases afterward. In Figure 6d, the dielectric coefficient of PZ8T2 gradually increases with rising temperature, reaching its maximum value near the *T_c_* (300 °C). Beyond the *T_c_*, the dielectric coefficient decreases due to a phase transition from a ferroelectric to a paraelectric state. The *T_c_* values of the three compositions closely match the values predicted by the phase diagram, demonstrating that the experimentally implemented compositions successfully reflect their thermal stability and structural characteristics. The dielectric coefficients at each *T_c_* exhibit distinct characteristics that align closely with the predictions of the phase diagram. The dielectric loss was transformed using a logarithmic scale, which exaggerated small variations, making minor fluctuations more pronounced. However, the loss trends consistently follow the behavior of the dielectric coefficient for each PZT composition, with a noticeable increase beyond *T_c_*, where losses become more prominent.

**Figure 7:**
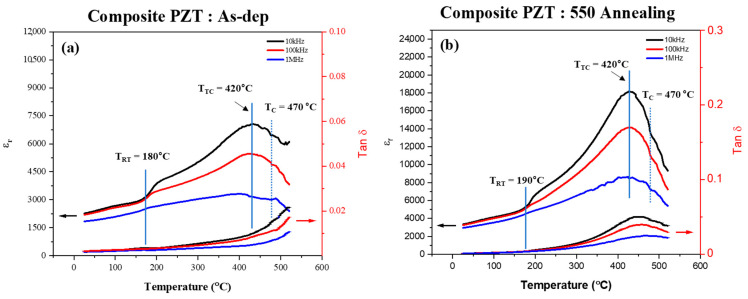
Dielectric coefficient and dielectric loss of composite AD PZT film in the (**a**) as-deposited and (**b**) annealed at 550 °C.

Figure 7 compares the dielectric coefficient (*ε_r_*) and dielectric loss (*tan δ*) of the composite PZT films before and after annealing at 550 °C, showing how they change as a function of temperature.

As shown in Figure 7a, the dielectric coefficient of the composite PZT AD thick film was implemented by maintaining the three compositions, and the phase-transition regions appeared according to the temperature range. This resulted in a relatively gradual increase in the dielectric coefficient from room temperature to 180 °C. For typical PZT, the dielectric coefficient is approximately 1300 at 25 °C and increases to 1500 at 198 °C, reflecting a dielectric coefficient change of about 15% over this temperature range [2]. In contrast, the composite PZT AD thick film demonstrated exceptional dielectric properties compared to typical bulk PZT. At 25 °C, the dielectric coefficient reached ~2271, nearly 75% higher than the 1300 reported for PZT. This trend continued at elevated temperatures, with the dielectric coefficient peaking at ~3359 at 178 °C. Within the temperature range of 25 °C to 178 °C, the permittivity increased by 48% at 10 kHz, 47% at 100 kHz, and 40% at 1 MHz. These results clearly demonstrate the film’s ability to maintain strong dielectric performance across a broad frequency range for a wide temperature range.

A key difference between conventionally processed PZT and AD-fabricated composite PZT is their behavior at elevated temperatures. Conventional bulk PZT exhibits a sharp phase transition near its Curie temperature, leading to a sudden and significant drop in dielectric permittivity. This instability arises due to the rapid transformation to the cubic phase, which disrupts its ability to maintain high dielectric performance at elevated temperatures. In contrast, the composite PZT AD thick film exhibits a more gradual phase transition, allowing for a smoother permittivity change. This characteristic enhances its suitability for high-temperature applications, as it mitigates abrupt dielectric variations and maintains performance over a wider operational range.

As shown in Figure 7b, annealing significantly enhanced the dielectric properties of the composite PZT film. For instance, at 25 °C, the dielectric coefficient increased by approximately 48% after annealing, and at 178 °C, the increase was about 59%. This indicates that annealing effectively increases the permittivity of the material across a wide temperature range. The annealing process significantly increased the dielectric coefficient of the composite PZT film, with changes exceeding the typical range observed in similar materials. This enhancement demonstrates the effect of annealing on optimizing the material’s dielectric properties across a wide temperature range. When annealed at 550 °C, the dielectric coefficient of the PZT film increased, but the dielectric loss also became larger. At high frequencies, increased dielectric loss can lead to significant power losses, which may be a limitation in continuous power applications. However, in pulse power applications, where large amounts of power are required for only a few seconds, such power losses are less critical [6,12]. Therefore, despite the increase in dielectric loss, AD-fabricated composite PZT films could be suitable for these applications, as they provide enhanced dielectric performance and effectively handle brief, high-power demands.

The single-phase PbZr_0.5_Ti_0.5_O_3_ film exhibited abrupt changes, with dielectric coefficient fluctuations of 86% and 107% between 200 °C and 300 °C, highlighting its instability [2]. The composite film, on the other hand, showed a slow, non-steep increase in permittivity over the same temperature range. At 10 kHz, the dielectric coefficient after 550 °C annealing was 6713 at 200 °C and increased to 10,410 at 300 °C, representing an increase of approximately 55%. Furthermore, at 350 °C, the dielectric coefficient reached 13,386, indicating an additional 28% increase from 300 °C to 350 °C, indicating that the composite PZT film facilitates smoother phase transitions, significantly enhancing the dielectric stability against temperature changes. This increase demonstrates the composite film’s ability to suppress abrupt phase transitions, ensuring consistent performance over a wide temperature range. These variations indicate the instability of conventional PZT as the transition to the cubic phase begins, leading to an increased rate of change in permittivity with temperature above 200 °C.

Based on Figure 6 and Figure 7, the dielectric properties of the composite PZT film and single-phase PZ5T5 film were analyzed to evaluate their temperature stability. For the PZ5T5 film, the dielectric constant in the low-frequency region was approximately 8490 at 200 °C and increased to about 12,495 at 420 °C, showing a variation of approximately 47.1%. In the high-frequency region, the dielectric constant increased from approximately 8183 at 200 °C to 10,502 at 420 °C, corresponding to a 28.4% increase. In contrast, the composite PZT film exhibited a dielectric constant of approximately 3904 at 200 °C and 6997 at 420 °C in the low-frequency region, reflecting an increase of 79.2%. In the high-frequency region, the dielectric constant increased from about 2663 at 200 °C to 3269 at 420 °C, indicating a 22.8% change. Although the composite PZT film displayed a higher rate of change in the low-frequency region, this was due to the relative change based on its lower initial dielectric constant. In terms of absolute values, the PZ5T5 film maintained a higher dielectric constant across all measured temperatures.

These findings indicate that while PZ5T5 maintains a higher overall dielectric constant, the composite PZT film exhibits reduced dielectric variation with temperature, demonstrating enhanced thermal stability. This is particularly notable in high-frequency applications, where stability near the Curie temperature (*T_c_*) is critical. Annealing at 550 °C significantly improved the crystallinity of the composite PZT film, leading to a substantial increase in permittivity. Moreover, in the temperature range just before the complete transition to the cubic phase, approximately between 400 °C and 430 °C, the permittivity distribution became more gradual. This effect was especially prominent in the high-frequency region, where the permittivity remained relatively stable near *T_c_*, highlighting the potential for high-temperature applications. These observations suggest that the composite PZT film, despite its initially lower dielectric constant, offers stable dielectric performance across a broad temperature range, making it a promising candidate for applications requiring high-temperature dielectric stability.

**Figure 8:**
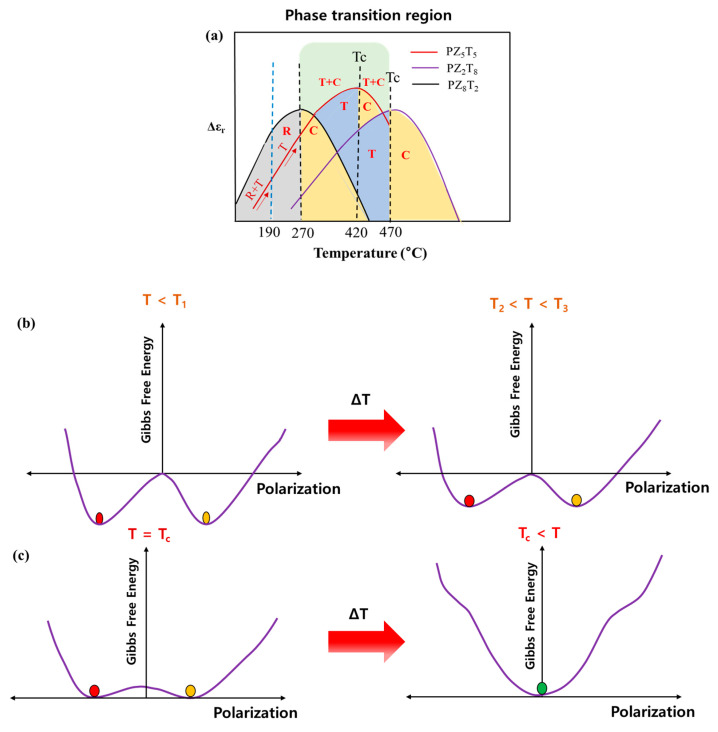
(**a**) Temperature-dependent phase coexistence and dielectric behavior of PZT compositions near the MPB and T_c_, where red, green, and yellow circles represent different crystal phases. (**b**) Gibbs free energy changes during the phase transition from phase coexistence to the tetragonal dominance phase, and (**c**) Gibbs free energy changes during the phase transition from tetragonal dominance to the onset of cubic phase transition [14].

Figure 8a illustrates the dielectric behavior of the composite PZT film as a function of frequency across a temperature range from room temperature to 530 °C. In particular, the temperature range from 190 °C to 420 °C, where a rapid change in dielectric coefficient occurs due to the onset of phase transitions, is further detailed in the figure on the right. This depiction clearly visualizes the phase-coexistence regions of the three compositions, allowing for a better understanding of the structural changes in each composition. When the temperature exceeds 190 °C, the PZ5T5 composition begins to shift away from the MPB region, with the tetragonal phase becoming increasingly dominant. This phase coexistence and transition enhance the dielectric coefficient in this temperature range. When the temperature reaches 270 °C, the PZ8T2 composition reaches its *T_c_*, marking the disappearance of the rhombohedral phase and the coexistence of tetragonal and cubic phases. This phase evolution further contributes to the dielectric response, maintaining stability across compositions. At 420 °C, the PZ5T5 composition reaches its *T_c_*, while the dielectric coefficient begins to decrease as the cubic phase becomes dominant. Finally, at 470 °C, the PZ2T8 composition transitions fully into a stable cubic phase, leading to a sharp drop in the dielectric coefficient due to the loss of ferroelectric properties. This sequence of overlapping phase transitions in the three compositions ensures stable and enhanced dielectric properties over a wide temperature range, highlighting the role of phase coexistence in maintaining consistent performance [13].

Figure 8b illustrates the phase-transition process in PZT with a single composition near the morphotropic phase boundary (MPB). At T_1_ (~200 °C), the phases coexist (rhombohedral and tetragonal), contributing to the overall dielectric response. The Gibbs free energy landscape exhibits two distinct local minima, representing the stability of the two coexisting phases. As T_2_ (the temperature increases beyond T_1_) starts, the overlapping phases are reduced by one, and the one phase (tetragonal) becomes dominant [12].

Figure 8c illustrates the phase-transition process in PZT with a mixed composition. In this case, the phases coexist (tetragonal and cubic phases), and the energy wells are smoothly connected, allowing the phase transition to proceed gradually. A significant increase in permittivity accompanies this structural transition, as the Gibbs free energy function transitions to a single deep minimum, indicating the stabilization of the tetragonal phase. As the material approaches T_3_ (~420 °C), the energy barrier between the tetragonal and cubic phases decreases, allowing for a gradual transition. The system reaches its peak permittivity before undergoing a final phase transition to the cubic phase. Beyond T_4_ (~420 °C and above), the Gibbs free energy function has only one global minimum at P = 0, corresponding to the paraelectric cubic phase, marking the loss of spontaneous polarization and a rapid decrease in permittivity as the material fully transitions into its paraelectric state [14].

To clearly describe the sequential phase transitions in the composite PZT film, we utilize the Gibbs free energy function expressed as follows [12,15]:

$$G\left(P\right)=\frac{1}{2}\;\alpha P^{2}+\frac{1}{4}\;\beta P^{4}+\frac{1}{6}\;\delta P^{6}$$

where *α* is the temperature-dependent coefficient governing phase stability, *β* is a positive constant ensuring stability, *P* is the polarization and *δ* accounts for higher-order polarization contributions. The parameter *α* varies with temperature as follows:

$$\alpha =A(T-T_{c})$$

such that it is negative in the ferroelectric phase, zero at the transition temperature, and positive in the paraelectric phase. At T_1_ (~200 °C), the rhombohedral (R) and tetragonal (T) phases coexist, contributing to the dielectric response. The Gibbs free energy function exhibits two distinct local minima, corresponding to the stability of these phases. The condition for equilibrium in this phase coexistence is derived from the following:

$$\frac{\mathrm{dG}}{\mathrm{dP}}=\alpha P+\beta P^{3}+\delta P^{5}=0$$

which leads to the existence of multiple nonzero polarization states. As the temperature increases to T_2_ (200~400 °C), the rhombohedral phase fraction diminishes, and the tetragonal phase dominates. The Gibbs free energy function stabilizes into a single deep minimum at the following:

$$P=\pm \sqrt{\frac{-\alpha }{\beta }}$$

indicating a unique ferroelectric phase. Approaching T_3_ (~420 °C), the Gibbs free energy barrier between the tetragonal and cubic states lowers, allowing for a smoother phase transition. Near this temperature, the Gibbs free energy takes the following form:

$$G\left(P\right)=\frac{1}{4}\;\beta P^{4}+\frac{1}{6}\;\delta P^{6}$$

which suggests the impending loss of polarization stability. At T_4_ (~420 °C and above), the system undergoes a complete transition to the paraelectric cubic phase, with Gibbs free energy reducing to a single global minimum at *P* = 0, satisfying the following:

$$G\left(P\right)=\frac{1}{2}\;\alpha P^{2}+\frac{1}{4}\;\beta P^{4}+\frac{1}{6}\;\delta P^{6}\;(\alpha >0)$$

**Figure 9:**
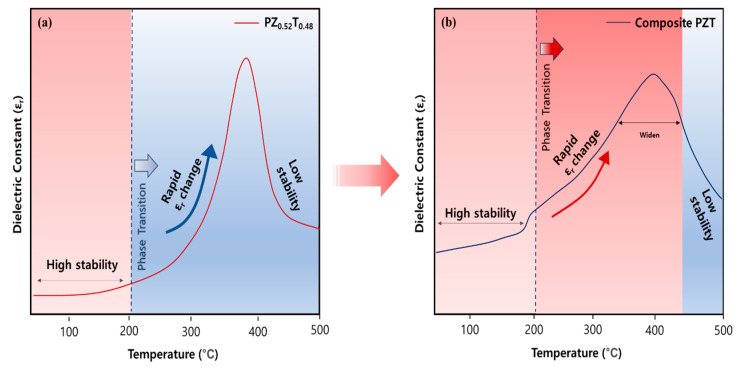
Comparison of temperature-dependent dielectric and loss properties of (**a**) single phase of PZT [2] and (**b**) composite PZT annealed at 550 °C.

The dielectric constants of single-phase PZT, shown in Figure 8a, and the composite PZT film, shown in Figure 8b, were compared to evaluate their performance in utilizing the phase-coexistence region for broader temperature stability. As shown in Figure 9, introducing compositions with different T_c_ through the AD process stabilizes the dielectric constant within the practical temperature range, from room temperature to approximately 400 °C. In the case of conventional single-phase PZT, a dielectric constant of around 1500 was achieved in the range from room temperature to about 200 °C [2]. However, by mixing PZT compositions, the coexistence region of rhombohedral and tetragonal phases was expanded, resulting in an increased dielectric constant of around 3000. In the temperature range where phase transitions begin, an analysis of the permittivity revealed that the composite PZT film exhibited an increase of approximately 55% between 200 °C and 300 °C and about 29% between 300 °C and 350 °C. This indicates that the dielectric constant of the composite PZT film shows a more gradual change across these temperature ranges, demonstrating enhanced thermal stability. In contrast, conventional single-phase PZT exhibited much larger fluctuations, with change rates of 87% from 200 °C to 300 °C and 107% from 300 °C to 350 °C. These results highlight the superior performance of the mixed PZT composition, particularly its ability to maintain more stable dielectric properties over a broader temperature range by enabling smoother phase transitions and reducing the temperature dependence of the dielectric constant [11,16,17,18,19,20].

## 4. Conclusions

Using the AD process, we successfully fabricated composite PZT film with three different phase-transition temperatures. The AD process enables the formation of a dense and uniform microstructure, which can contribute to maintaining a stable, high dielectric coefficient near T_c_. Additionally, the AD process contributed to an overall increase in the dielectric coefficient of the composite film, further enhancing its performance across a wide temperature range. The instability in the dielectric coefficient observed in single-phase PbZr_0.5_Ti_0.5_O_3_ due to rapid phase transitions above 200 °C was effectively resolved, ensuring a relatively stable dielectric coefficient within a temperature range of up to 400 °C.

While the single-phase PbZr_0.5_Ti_0.5_O_3_ film exhibited abrupt changes, with fluctuations of 86% and 107% between 200 °C and 300 °C, the composite films showed s dielectric coefficient that increased by approximately 55%, while between 300 °C and 350 °C, it increased by about 28%. This gradual change indicates that the composite PZT film facilitates smoother phase transitions, significantly enhancing thermal stability. These sharp variations highlight the instability of conventional PZT caused by rapid phase transitions above 200 °C.

## Figures and Tables

**Table 1:** Condition of Typical Aerosol Deposition Method.

AD Conditions	
Carrier gas	N_2_
Size of the nozzle orifice	5 × 1 mm^2^
Pressure in the deposition chamber	0.060 torr
Mass flow (MFC)	9~17 L/min
Scanning speed of the substrate	0.8~1 mm/sec
Distance between the nozzle and substrate	1~4 mm

**Table 2:** P_r_, P_s_, and P_max_ values for PZ5T5, PZ2T8, PZ8T2, and the composite PZT compositions.

Composition	P_r_ (µC/cm^2^)	P_s_ (µC/cm^2^)	P_max_ (µC/cm^2^)
PZ8T2	19.61	37.47	58.08
PZ2T8	16.13	35.55	127.79
PZ5T5	28.20	57.77	46.25
Composition PZT	32.91	47.44	131.51

## Data Availability

The original contributions presented in the study are included in the article, and further inquiries can be directed to the corresponding author.
